# High-Frequency Heart Rate Variability Linked to Affiliation with a New Group

**DOI:** 10.1371/journal.pone.0129583

**Published:** 2015-06-24

**Authors:** Baljinder K. Sahdra, Joseph Ciarrochi, Philip D. Parker

**Affiliations:** Institute for Positive Psychology and Education, Australian Catholic University, Strathfield, New South Wales, Australia; Bournemouth University, UNITED KINGDOM

## Abstract

This study tests the hypothesis that high levels of high-frequency heart rate variability (HF-HRV) predisposes individuals to affiliate with new groups. Resting cardiac physiological recordings were taken before and after experimental sessions to measure trait high-frequency heart rate variability as an index of dispositional autonomic influence on heart rate. Following an experimental manipulation of priming of caring-related words, participants engaged in a minimal group paradigm, in which they imagined being a member of one of two arbitrary groups, allocated money to members of the two groups, and rated their affiliation with the groups. High levels of HF-HRV were associated with ingroup favouritism while allocating money, an effect largely attributable to a positive relationship between HF-HRV and allocation of money to the ingroup, and less due to a negative relationship between HF-HRV and money allocation to the outgroup. HF-HRV was also associated with increased self-reported affiliation feelings for the ingroup but was unrelated to feelings towards the outgroup. These effects remained substantial even after controlling for age, gender, BMI, mood, caffeine consumption, time of day of data collection, smoking and alcohol behaviour, and respiration rate. Further, the effects were observed regardless of whether participants were primed with caring-related words or not. This study is the first to bridge a long history of research on ingroup favouritism to the relatively recent body of research on cardiac vagal tone by uncovering a positive association between HF-HRV and affiliation with a novel group.

## Introduction

Human beings are fundamentally social. Are there biological factors that predispose us to affiliate with groups? One likely candidate is the cardiac vagal tone or the autonomic influence on heart rate. A healthy heart has a fluctuating rhythm. The normal variability in heart rate occurs, in part, from the synergistic actions of the sympathetic and parasympathetic branches of the autonomic nervous system (ANS), which allow the cardiovascular system to adapt to varying internal biological conditions as well as external situations [1, 2]. Fluctuations in heart rate that occur at the high frequency (0.15 to 0.4 Hz) range are considered to clearly represent the chronotropic effect of the parasympathetic nervous system on the heart via the vagus (10^th^ cranial) nerve, and are linked to Respiratory Sinus Arrhythmia (RSA), the increase and decrease in heart rate with inhalation and exhalation, respectively [1, 3, 4]. High-frequency heart rate variability (HF-HRV), as measured under resting conditions, represents a relatively stable autonomic disposition [5, 6].

There is a growing body of evidence linking HF-HRV with individual differences in regulation of emotions and cognitive functions: High HF-HRV is linked to positive emotions and interpersonal connectedness [7–9], stability of positive affect in daily life [10], better emotional responding and affect regulation [11, 12], greater cognitive performance [6, 13], better attention regulation [14], better impulse control [15], and better self-regulation [16]. To the extent that HF-HRV can be interpreted as a trait of ANS response to the environment [5], it might also be linked to social adaptability in a situation where people have a chance to affiliate with new groups. This makes sense theoretically because the “vagal brake” on heart rate allows efficient use of glucose to meet the metabolic costs of social information processing [17, 18], and flexible redirecting of energy from the periphery to the brain to better attend to situational cues [5, 6]. Based on these theoretical perspectives and empirical studies showing positive relationship between cardiac vagal tone and various aspects of socio-cognitive functioning, one can expect a link between HF-HRV and individual differences in the ease with which people can affiliate with novel groups.

People are generally motivated to identify themselves with social groups, often derive their self-regard at least partly from their group memberships, and tend to favour the groups to which they belong (ingroups) compared to the groups to which they do not belong (outgroups) [19]. A mere categorization of the self as a member of a totally new group about which one knows very little and has not even met any other member of that group, can be enough to elicit ingroup favouritism. In a typical minimal group paradigm, participants are randomly assigned to one of two groups based on an arbitrary criterion (e.g., colour of shirts, preferences for art work) and later asked to evaluate or allocate money to members of the two groups. People tend to favour their own newly formed group at the expense of the other group [20–22]. It is noteworthy that ingroup favouritism is not the same as outgroup derogation [23, 24], which can involve holding negative attitudes towards the outgroup or actively harming outgroup members. The minimal group paradigm has been well-studied for over 40 years and the phenomenon of ingroup favouritism in minimal groups is among the most robust findings in social psychology [20, 22, 25, 26].

The current study aims to bridge the long history of research on social identity in minimal groups to the relatively recent line of research on cardiac vagal tone. HF-HRV in particular is an interesting variable from a social psychological perspective. Autonomic reactions of the body, mediated by cortical and subcortical pathways, are important for social information processing and flexible allocation of energy to the brain and the periphery [18, 27]. In a socially threatening or unsafe situation, the influence of the vagus nerve on the body is expected to be minimized or shut down to redirect metabolic resources for a flight/fight response; whereas in a relatively safe social situation, the influence of the vagus is expected to be increased or maintained to promote calm behavioural states, self-regulation and prosocial/affiliative responses [18, 28]. Social psychological studies have found that cardiac vagal tone is negatively associated with attachment anxiety and perceived lack of security in close relationships [29], and marital conflict [30]; and positively associated with perceived social support [31], positive emotions and feelings of connectedness during social interactions [9], having more supporting friends [32], and coping with sadness with higher social engagement [33]. The current study focuses on a relative safe context of minimal groups, in which cardiac vagal tone is expected to be linked to affiliation with the new group.

Specifically, this study tests the hypothesis that HF-HRV would be linked to ingroup favouritism in a minimal group context such that it would be easier for people with high resting HF-HRV, compared to those with low resting HF-HRV, to affiliate with the minimal ingroup and allocate money to the ingroup compared to the outgroup. The study also included a priming manipulation of caring-related words to test whether caring priming might boost ingroup favouritism by increasing caring for the ingroup or buffer against ingroup favouritism by extending caring feelings towards the outgroup, and to test whether HF-HRV might moderate the effects of priming. Finally, we examined participants’ current mood at the outset to test whether current mood might moderate observed effects.

## Method

### Ethics Statement

All participants provided written informed consent. The study had full ethics clearance from the University of Western Sydney’s Human Research Ethics Committee (H9798).

### Participants

Ninety-one individuals (71% female) participated in the study. Of the participants, 88% were undergraduates and the remaining 12% were local community members recruited through study flyers distributed on and around campus. Undergraduates recruited from the psychology subject pool received course credit, and other participants received AU $20 per hour remuneration. Participants belonged to a diverse range of ethnicities: 35% of European descent, 27% from North-Africa/Middle-East, 12% from Asia, 3% from America, 2% from Oceania, 1% from Sub-Saharan Africa, and the remaining 20% from mixed ethnic backgrounds. Regarding their education level, 35% had completed high school, 20% vocational training, 12% advanced diplomas, 24% undergraduate degree, 4% graduate diploma and 5% postgraduate degree. Regarding annual household income, 33% reported under AU $30,000, 23% stated $30,000-$60,000, 22% stated $60,000-$90,000, and 20% reported annual household income higher than $90,000. Age ranged from 18 to 55 (*M* = 25.33, *SD* = 9.33).

### Procedure

An experimenter was present in the lab room at all times sitting on a separate desk with her back towards participants. Other than a brief interaction to obtain written informed consent and an initial 1-min test physiological recording for setting up the equipment, participants did not interact with the experimenter while completing the study tasks, all of which were automatically administered on a computer. Participants wore headphones throughout the session and received audio instructions for the computer-administered tasks. The following measures were taken:

### High-frequency heart rate variability (HF-HRV)

Each experiment session included two 6-min resting physiological recordings at the beginning and end of the session. Respiration and ECG Lead II recordings were taken using the Biopac MP150 data acquisition system and a dual wireless respiration and ECG BioNomadix module consisting of a transmitter and receiver. The sampling rate was 1000 Hz. During each resting recording, participants were asked to sit in a comfortable position in a chair and try to refrain from moving while resting their gaze on a fixation cross (+) displayed in the center of the computer screen in front of them.

No instructions were provided for breathing because the normal vagal influence on the heart is best interpreted during spontaneous breathing [1, 34]. For ECG recordings under resting conditions, HRV uncorrected for respiration is usually a reliable indicator of cardiac vagal tone, but some researchers have argued that correcting for respiration can substantially affect associations of HRV with other variables [35–37]. Measurement of respiration rate alongside ECG recordings allowed us to examine the correlations between HF-HRV and other variables with and without controlling for respiration.

Participants with a cardiac or neurological illness and those taking medications that can affect the heart function (e.g., beta blockers, opioid agonists) were excluded from HF-HRV analysis because it is difficult to make clear inferences about the normal vagal innervation on the heart when medication or pathology related factors are at play [1, 2]. Further exclusion criteria included equipment malfunction or respiration rate outside the range within which HF-HRV is interpretable (0.15–0.4 Hz) [1, 4]. The final sample of ECG recordings consisted of pre and post resting recordings from 75 participants: 73% female; age ranged from 18 to 55 (*M* = 24.45, *SD* = 8.55).

Raw ECG recordings of these participants were processed using the guidelines of the *Task Force of the European Society of Cardiology and the North American Society of Pacing and Electrophysiology* [4]. Kubios HRV 2.1 (Biosignal Analysis and Medical Imaging Group, University of Kuopio, Finland) was used for pre-processing and power spectral analysis [38]. The first and last 30 seconds of each recording were trimmed to remove artifacts introduced by setting-related reasons (e.g., experimenter starting recording, large movements due to posture adjustments by participants at the beginning or end of recording), leaving the relatively clean middle 5-min section of each recording. The RR-time series were automatically detected using Kubios QRS detection algorithm, which uses bandpass filtering and moving average filtering, followed by adaptive decision rules. The RR-time series were then processed using automatic artifact detection and detrending (using smoothn priors, λ = 500), and cubic spline interpolation to replace automatically detected artifacts. A trained research assistant manually inspected each recording to confirm automated artifact correction and manually correct for missed artifacts when necessary. Overall, the rate of erroneous beats in the raw 5-min ECG recordings was generally low (ranging from 0 to 5%).

The pre-processed RR-time series were subjected to frequency domain analysis. Cubic spline interpolation at the sampling rate of 4 Hz (default in Kubios 2.1) was used to convert the RR-time series into equidistantly sampled series prior to spectrum estimation. Using the Welch’s periodogram method, the RR-time series was divided into overlapping segments (50% overlap), which were windowed (256s width). The Fast Fourier transform spectra of these windows were averaged to yield spectrum estimates. We focused on the absolute power (ms^2^) of the power spectrum density in the high frequency (0.15–0.4 Hz) band because this component of the power spectrum reflects parasympathetic influence on the heart and is related to RSA [1, 3]. In contrast, fluctuations in the low frequency band are difficult to cleanly attribute to any single branch of the ANS because they reflect both sympathetic and parasympathetic activity and possibly other factors such as diurnal variations [1, 4].

### Priming

Right after the initial resting physiological recording, we employed a priming task adapted from the loving-kindness method used in previous research [39]. Participants were randomly assigned to one of three priming conditions. In the caring priming condition (*n* = 25), participants engaged in a brief (3.24 min) audio-guided loving-kindness meditation. They were asked to bring to mind a stranger they may have come across recently but did not know personally, and extend caring feelings towards this stranger. Several caring-related words were used throughout the instructions to prime caring: compassion, warmth, generosity, kindness, respect, considerate, gentleness, and goodwill. Typical loving-kindness meditation phrases (e.g., “May this person be free of suffering and hurt. May this person be well, safe, happy”) were also used. In the control condition (*n* = 24), participants received audio instructions to engaged in a brief (3.26 min) guided visualization of facial features of a stranger they came across recently but did not know personally. There was no reference to caring-related words. Instead, participants were guided to draw attention to each of the facial features of the stranger. This control condition was designed to control for the cognitive factors engaged in the caring condition (e.g., attention and working memory) but without the affective caring component. In the baseline condition (*n* = 26), participants did not engage in any guided visualization exercise.

### Minimal group categorization

To induce minimal group identity in the laboratory, participants received the following instructions, which were similar to the imagination instructions used in previous research [40]:

A small number of people in Sydney have been divided into two groups based on their preferences regarding two art styles. Half of the people were put into the Red group based on their liking of a particular kind of art. The remaining half were put into the Blue group based on their liking of a different kind of art. Imagine that you have been randomly assigned to the Blue group. Please memorize the name of your group.

To check whether participants understood their minimal group identity, on the next screen, participants were asked to type the name of the group to which they belonged (Blue group in all cases).

### Allocation of money to ingroup and outgroup members

Participants then received the following instructions before allocating money to the Blue and Red group members:

There are approximately 90 people responding to this survey. Imagine that about half the people like you belong to the Blue group, while the others belong to the Red group. The next phase of the survey asks respondents to allocate money to other members of these groups. Since participation in this study is anonymous, we assign arbitrary numbers to respondents. You are Member 47. You will be asked to imagine allocating money to 6 members of the Blue group and 6 members of the Red group, all of whom will be identified by numbers.

On the next screen, participants were shown a picture of Australian currency of various denominations, and asked to take a moment and image allocating money to the Blue and Red group members. Participants then received allocation matrices originally developed by Tajfel and colleagues [20], and subsequently validated in many studies [25], that pull for several possible allocation strategies: fairness or parity, maximum joint profit, maximum difference in favour of the ingroup, absolute ingroup profit, and maximum joint profit. Technical details of the design of Tajfel allocation matrices are thoroughly described elsewhere [26]. Each of the six matrices we used contained 2 rows and 14 columns. The first column contained information about the member number and group colour (e.g., Member 5 of the Blue group, Member 31 of the Red group). The remaining matrix contained different dollar amounts participants could assign to the Blue and Red member in that matrix. The order of presentation of the matrices was randomized for each participant. To keep participants’ own group identification salient, on the top right corner of each screen containing any given matrix, participants’ own member number and group was mentioned (Member 47 of Blue Group).

### Self-reported feelings towards the two groups

In a measure adapted from previous research [40], participants were asked to rate their agreement on a 7-point scale (1 = *strongly disagree* to 7 = *strongly agree*) with eight items, four per group (e.g., “I like the Blue group”, “The Red group is good,” “I feel attached to the Blue group,” “I identify with the Red group”). Cronbach’s alpha was .85 for the Blue group items and .80 for the Red group items.

### Potential control variables

Mood was assessed at the outset before the first resting physiological recording and again before the second resting recording. Participants were asked to describe their current mood using a scale ranging from 1 (*Extremely bad mood*) to 4 (*Neutral*) to 7 (*Extremely good mood*). Self-reported height and weight was used to calculate body mass index (BMI). To evaluate caffeine consumption prior to the experiment, participants were asked to indicate the number of cups of drinks they had of coffee, tea or other caffeinated drinks. Participants were also asked to characterize their smoking behavior using the following scale: 1 (*Never smoked*), 2 (*Ex-smoker*) and 3 (*Current smoker*). To measure general alcohol consumption, participants were asked to rate on the following scale: 1 (*0 standard drinks per week*), 2 (*1 to 5 standard drinks per week*), 3 (*5 to 10 standard drinks per week*), and 4 (*More than 10 standard drinks per week*).

## Results

Analyses were conducted in the statistical program R [41]. Preliminary analyses included a general linear model using natural log transformed HF-HRV values from pre and post study resting recordings as within-subjects variable and priming as between-subjects variable. On average, post study HF-HRV level was slightly lower (*M* = 5.62, *SD* = 1.09) than pre study HF-HRV (*M* = 5.79, *SD* = 1.17), *F*(1, 72) = 4.17, *p* = .05. There was no main effect of priming or moderation of priming by HF-HRV (*F*s<1). The pre to post difference in HF-HRV was eliminated, however, when age was added as a control variable, *F*(1, 71) = 1.16, *p* = .29. We therefore report correlations of other variables with HF-HRV separately for pre and post recordings both with and without controlling for age and other variables that can potentially affect HRV, including gender, BMI, time of the day the ECG was taken, mood, use of caffeine prior to assessment, general smoking and alcohol behaviour, and respiration rate. We also computed a mean HRV score in keeping with the recommendations to aggregate across measurements to derive trait-level HF-HRV [5], and examined both zero-order and partial correlations of the mean HRV with other variables. Point estimates and 95% confidence intervals (CIs) of means and correlations between variables reported below were computed using the bias-corrected-and-accelerated (BCa) bootstrap procedure. This method was preferred because, unlike parametric CIs, bootstrapping is robust to violations of normality [42, 43].

### Ingroup favouritism (IF) in allocation matrices

The allocation matrices were analyzed using a method similar to the one employed in previous research [44], by totaling the money allocated to the ingroup (Blue group) and outgroup (Red group) from all the matrices, and computing the difference between these two to derive a single index of ingroup favourtism (IF). Money allocations to the ingroup (Blue group) and the outgroup (Red group) were orthogonal: the BCa bootstrapped correlation was -0.09, 95% CI [-0.48 0.33]. However, on average, participants allocated more money to the ingroup than outgroup: The BCa bootstrapped estimate of the mean difference was 9.83, 95% CI [5.99 15.52]. This finding replicates the classic ingroup favouritism effect well-documented in social psychology [22].

### HF-HRV and IF

**Fig 1:**
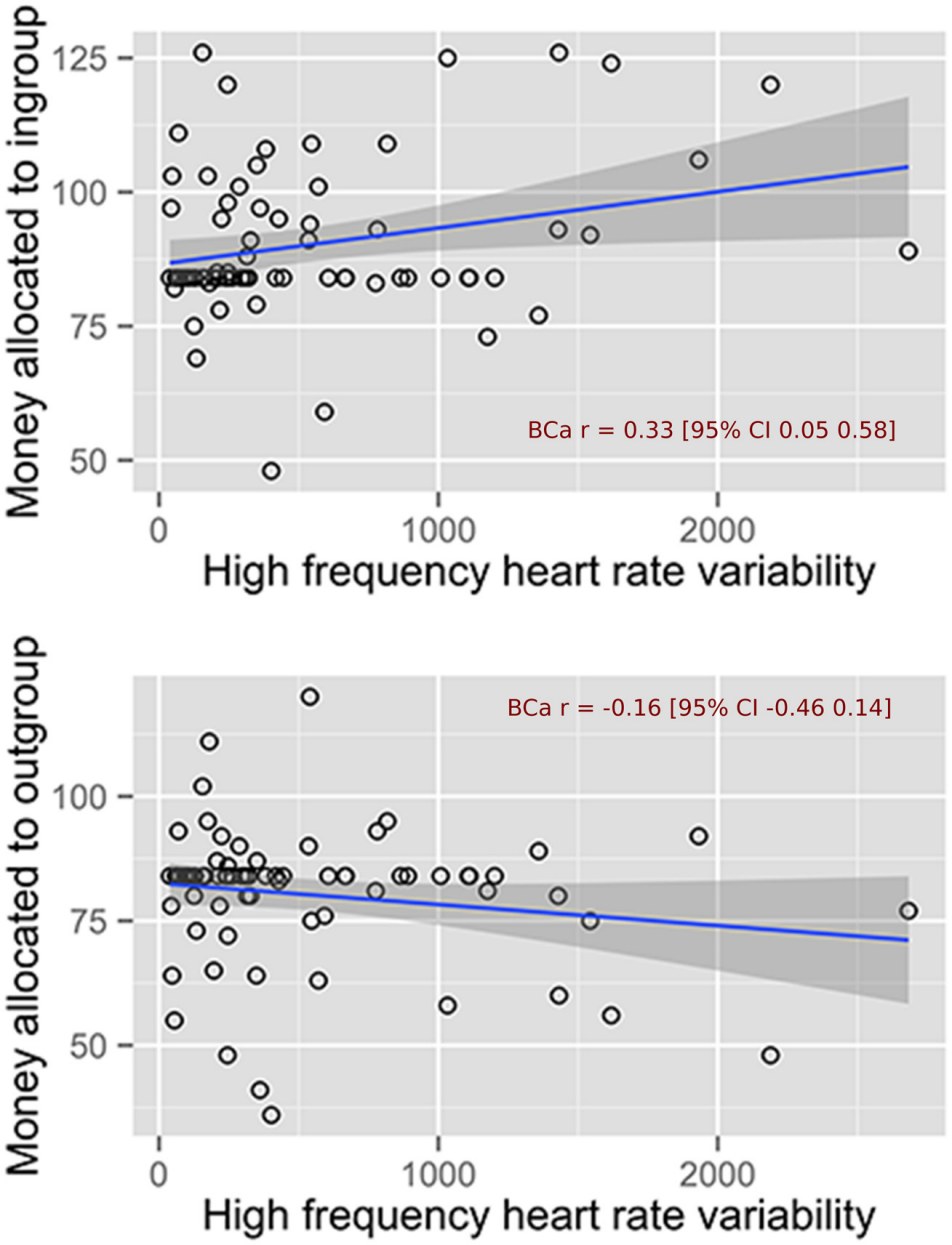
Scatterplots with lines of best fit and 95% confidence internals demonstrating the association between mean HF-HRV and money allocation to the ingroup (top panel) and the outgroup (bottom panel).

Table 1 contains the BCa bootstrapped correlations and 95% CIs of zero-order and partial correlations of the variables derived from the Tajfel matrices and HF-HRV from the two resting recordings and the mean HF-HRV. The results consistently demonstrate that HF-HRV was positively related to IF for pre and post study recordings as well as the mean HF-HRV. These effects are largely attributable to the positive associations of HF-HRV with money allocation to the ingroup rather than the outroup. The correlations of HF-HRV variables with money allocation to the ingroup were all substantial whereas those with money allocation to the outgroup were negligible. This pattern of results remained substantial even after controlling for age, gender, BMI, time of day of data collection, mood at the time of measurement, caffeine consumption prior to assessment, general smoking and alcohol behaviour and respiration rate. In short, in the context of minimal group paradigm, high HF-HRV was linked to genuine ingroup favouritism and not necessarily outgroup derogation. Fig 1 illustrates the link between HF-HRV and money allocation to ingroup and outgroup. It contains scatterplots with lines of best fit and 95% confidence internals demonstrating the association between mean HF-HRV and money allocation to the ingroup (top panel of the figure) and the outgroup (bottom panel).

**Table 1:** The BCa bootstrapped estimates and 95% confidence intervals of zero-order and partial correlations of Tajfel matrices variables with HRV variables.

	Money allocation to ingroup	Money allocation to outgroup	Difference score of ingroup favouritism
**Pre HF-HRV:**			
Zero-order cor.	0.25* [0.005 0.50]	-0.13 [-0.38 0.10]	0.26* [0.001 0.51]
Partial cor.	0.30* [0.006 0.62]	-.010 [-0.40 0.18]	0.27* [0.06 0.61]
**Post HF-HRV:**			
Zero-order cor.	0.23* [0.03 0.45]	-0.20 [-0.45 0.05]	0.29* [0.01 0.53]
Partial cor.	0.28* [0.03 0.52]	-0.20 [-0.47 0.11]	0.32* [0.04 0.59]
**Mean HF-HRV:**			
Zero-order cor.	0.26* [0.002 0.49]	-0.17 [-0.42 0.08]	0.29* [0.01 0.52]
Partial cor.	0.33* [0.05 0.58]	-0.16 [-0.46 0.14]	0.33* [0.02 0.59]

*Note*. Partial correlations were conducted controlling for age, gender, BMI, time of the day the ECG was taken, mood, caffeine consumed prior to assessment, general smoking and alcohol behaviour, and respiration rate.

* *p* < .05.

### HF-HRV and self-reported feelings towards the group

Self-reported feelings toward the ingroup and those towards the outgroup were unrelated: the BCa bootstrapped correlation was -0.15 [-0.39 0.11]. Table 2 shows that HF-HRV variables were positively associated with self-reported affiliation feelings towards the ingroup but unrelated to feelings towards the outgroup. The pattern of results remained the same even after adding the control variables. This further supports the notion that high trait level cardiac vagal tone is conducive to affiliating with new groups.

**Table 2:** The BCa bootstrapped estimates and 95% confidence intervals of zero-order and partial correlations of self-reported affiliation feelings towards the two groups with HRV variables.

	Self-reported affiliation with ingroup	Self-reported affiliation with outgroup
**Pre HF-HRV:**		
Zero-order cor.	0.24* [0.020.43]	0.01 [-0.28 0.26]
Partial cor.	0.31* [0.10 0.52]	0.10 [-0.21 0.37]
**Post HF-HRV:**		
Zero-order cor.	0.19 [-0.03 0.42]	0.08 [-0.19 0.33]
Partial cor.	0.25* [0.01 0.50]	0.14 [-0.20 0.41]
**Mean HF-HRV:**		
Zero-order cor.	0.23* [0.004 0.44]	0.04 [-0.24 0.29]
Partial cor.	0.31* [0.05 0.55]	0.11 [-0.21 0.38]

*Note*. Partial correlations were conducted controlling for age, gender, time of the day the ECG was taken, mood, caffeine consumed prior to assessment, general smoking and alcohol behaviour, and respiration rate.

* *p* < .05.

### Cross-validation

We employed a 10-fold cross-validation method to ensure that our key findings were not biased by potentially influential data-points. A 10-fold cross validation method belongs to the class of resampling methods that employ machine learning principles to train a model on one or more samples and test its accuracy on a different sample. A 10-fold cross validation method randomly divides data into 10 subsets of approximately equal sizes. It then uses nine datasets for training, and one remaining dataset for testing prediction accuary of the model previously trained on nine datasets. It repeats this procedure ten times, and then computes the mean prediction accuracy. A 10-fold cross validation method provides the most optimal bias-variance trade-off and tends to give a more accurate estimate of test error rate than alternative methods, such as split-sample or leave-one-out cross-validation methods [45].

In our data, a 10-fold cross validation test of the link between mean HF-HRV and IF yielded a mean squared error of 0.95, which was virtually identical to the mean squared error of 0.96 of the original model using the entire sample in which z-scores of mean HF-HRV were used to predict z-scores of IF. Similarly, a 10-fold cross validation test of the link between mean HF-HRV and self-reported feelings towards the ingroup produced a mean squared error of 0.96, which was very close to the mean squared error of 0.98 of the original model based on the entire sample. These tests confirm that the estimates of the key findings reported above were likely unbiased by potentially influential data points.

## Discussion

We uncovered a link between HF-HRV, a noninvasive measure of parasympathetic nervous system innervation on heart rate, and individual differences in people’s tendencies to affiliate with and favour a minimal ingroup created in the laboratory. The finding helps us to connect the vast literature on minimal groups with a relatively recent growing literature on cardiac vagal tone. High levels of HF-HRV were associated with ingroup favouritism in money allocation matrices, an effect largely attributable to a positive relationship between HF-HRV and allocation of money to the ingroup, and less due to a negative relationship between HF-HRV and money allocation to the outgroup. High HF-HRV was also associated with increased affiliation feelings for the ingroup but was unrelated to feelings towards the outgroup. The results were consistent across both resting recordings and they held up even after controlling for age, gender, BMI, time of day of measurement, mood, caffeine consumed prior to assessment, general smoking and alcohol behavior and respiration rate. In sum, there was a greater tendency for people with high dispositional cardiac vagal tone to affiliate with a minimal group in the laboratory and allocate money to the ingroup members.

The effect of the minimal group paradigm appeared to be too potent to be shifted by a brief caring priming manipulation. Participants in the three priming conditions (no-priming baseline, facial features priming, and caring priming) did not differ in self-reported affiliation feelings towards the two groups, ingroup favouritism or trait HF-HRV. Simply put, participants generally cared for their ingroup more than they did for the outgroup, regardless of whether they were primed with caring-related words or not. This rather robust phenomenon of ingroup favouritism is consistent with over 40 years of research in intergroup processes [20, 22, 25, 46].

Note that the minimal group procedure that we used allowed us to induce minimal group identity and measure ingroup favouritism using the matrices without using any deception in the study design. In the classical minimal group paradigm, participants are ostensibly assigned to a new group based on their performance on a task; that is, participants are deceived into believing that they belong to a new group based on some supposedly meaningful criterion (e.g., rating a series of paintings, estimating number of dots on a screen). However, consistent with recent evidence [40], we found that asking participants to imagine belonging to an arbitrary group, which involves no deception, was effective in producing ingroup favouritism.

We did use the key features of the classical minimal group paradigm while measuring ingroup favouritism. Participants never allocated money to themselves in any of the matrices, and did not expect to have any interactions with the individuals to whom they allocated money, all of whom were identified by numbers. The only thing that the participants were certain of was the group membership of the individuals to whom they allocated money. This procedure arguably distills in the laboratory the intergroup processes that are purely due to categorization of the self into a group, and not due to personal self-interest or previously held beliefs or expectations about the ingroup and outgroup, which are common in groups with long histories [22]. The mere categorization of oneself as a member of an arbitrary Blue group in our study was enough to elicit affiliation with that group and ingroup favouring behavior, especially among those with high levels of dispositional cardiac vagal tone.

Future research is needed to replicate the effects reported here. Further, more work is needed to examine other biological systems that might also be relevant to affiliation with new groups. In research on close relationships, several biological systems, such as, the hypothalamic-pituitary-adrenal (HPA) axis of the endocrine system, oxytocin and vasopressin systems, and endogenous opioids and catecholamines, in addition to the ANS, are considered to be important for attachment processes in intimate relationships [47]. For instance, individuals who exhibit insecurity (anxious or avoidant attachment styles) in close relationships tend to show emotion regulation deficits, muted vagal innervation on the heart, and heightened HPA and ANS reactivity to stressors [48]. In our study, we used a safe, fairly neutral situation of a minimal group paradigm to test the link between cardiac vagal tone and affiliation with a social group with no intimate relationships. It remains to be seen how HPA and ANS reactivity to stressors in intergroup conflicts situations, for instance, might be linked to intergroup processes.

Future research should also examine the link between trait HF-HRV and intergroup processes in real groups with complex relationships (e.g., Indigenous Australians vs. White Australians; Arabs and Americans). The benefit of studying minimal groups, like the one we used in our study, is that they allow a clean attribution of outcomes to the mere categorization of the self as a member of the group. However, the link between cardiac vagal tone and group affiliation may be stronger in the context of real groups with long histories and deeper personal meanings for participants.

## Supporting Information

S1 Dataset Dataset of the study. (XLS)
